# New Application of Quartz Crystal Microbalance: A Minimalist Strategy to Extract Adsorption Enthalpy

**DOI:** 10.3390/nano12224035

**Published:** 2022-11-17

**Authors:** Zhiheng Ma, Tongwei Yuan, Yu Fan, Yang Chen, Yueling Bai, Zhixuan Cheng, Jiaqiang Xu

**Affiliations:** NEST Laboratory, Department of Physics, Department of Chemistry, College of Science, Shanghai University, Shanghai 200444, China

**Keywords:** quartz crystal microbalance (QCM), gas sensor, metal−organic frameworks (MOFs), CO_2_, adsorption enthalpy

## Abstract

The capture and separation of CO_2_ is an important means to solve the problem of global warming. MOFs (metal–organic frameworks) are considered ideal candidates for capturing CO_2_, where the adsorption enthalpy is a crucial indicator for the screening of materials. For this purpose, we propose a new minimalist solution using QCM (quartz crystal microbalance) to extract the CO_2_ adsorption enthalpy on MOFs. Three kinds of MOFs with different properties, sizes and morphologies were employed to study the adsorption enthalpy of CO_2_ using a QCM platform and a commercial gas sorption analyzer. A Gaussian simulation calculation and previously data reported were used for comparison. It was found that the measuring errors were between 5.4% and 6.8%, proving the reliability and versatility of our new method. This low-cost, easy-to-use, and high-accuracy method will provide a rapid screening solution for CO_2_ adsorption materials, and it has potential in the evaluation of the adsorption of other gases.

## 1. Introduction

With the continuous increase in human’s energy demand, fossil fuel resources are not only depleted day by day, but the greenhouse effect caused by the large amount of CO_2_ emissions from the combustion of fossil fuels has also given rise to serious environmental problems [1,2]. Global warming [3], sea level rise [4] and extreme climate change [5] caused by the greenhouse effect have also greatly threatened the sustainable development of human society. Therefore, it is urgent for human beings to reduce the emission of CO_2_ into the atmosphere and curb the greenhouse effect. On the one hand, human beings need to develop and use new energy to reduce CO_2_ emissions [6,7,8]. On the other hand, the amount of CO_2_ in the atmosphere needs to be reduced. Many scientists have committed to the reduction of CO_2_, including through the use of electrochemical [9] and photocatalytic methods [10]. However, large-scale direct capture of CO_2_ from the air and its use as raw material in chemical production, food and beverage manufacturing, agriculture and medicine is the most economical way [11,12]. Many materials have been proven to have great application potential in the separation, capture and solidification of CO_2_, such as zeolites [13], activated carbon [14] and organic solids [15], but these materials have some shortcomings that restrict their ability to adsorb CO_2_, for example, the specific surface area is not large enough, the pore size is fixed or it is difficult to graft the functional groups, which hinder the improvement of their CO_2_ adsorption capacity.

Metal–organic framework-based materials (MOFs) are a new type of porous material with a unique structure, large surface area, good chemical tunability and good stability. Recent studies have shown that MOF-based materials have excellent carbon dioxide capture capabilities [16,17,18]. To achieve the enrichment and reuse of CO_2_, we need to select suitable MOF materials by evaluating their adsorption enthalpy for CO_2_. According to classical physical–chemical adsorption theories [19,20], when the ΔH value is in the range of −40 kJ/mol–0 kJ/mol, the interaction between MOF materials and molecular CO_2_ is weak reversible physical adsorption. When the ΔH value is located in the range of −80 kJ/mol–−40 kJ/mol, the adsorption of CO_2_ onto the MOF materials is selective and reversible adsorption, which is suitable for gas sensing material. The desorption becomes hard when the adsorption enthalpy is lower than −80 kJ/mol, meaning that those materials could be used to solidify CO_2_. Therefore, the adsorption enthalpy is a crucial parameter for the evaluation of whether MOFs can be applied to CO_2_ capture or sensing material.

The measurement of the adsorption enthalpy is usually suitable for large commercial adsorption instruments, but it is expensive, cumbersome and requires professionals to operate. Although the data are accurate, the method is not convenient, and it is not conducive to further scientific research and industrialization processes. Herein, we first propose a minimalist strategy by using quartz crystal microbalance (QCM) to measure the enthalpy of CO_2_ adsorption onto the surface of MOFs. This method only needs to measure two sets of CO_2_ response curves at different temperatures over a short time.

In order to verify the accuracy of this novel method, data from a large commercial adsorption instrument (Micromeritics ASAP 2020 system) and a Gaussian calculation were collected for comparison; it was found that the error in the QCM platform was only 6.8%. Due to the high reliability, minimal operation and low costs, this method for the measurement of CO_2_ adsorption enthalpy has great potential to compensate complicated current technology. To verify the feasibility and universality of our method, we expanded this new method to ZIF-8, UiO-66 and MOF-74 with different sizes and morphologies. At the same time, in comparison with another method, the cantilever beam extraction of adsorption enthalpy, the QCM platform used in our method is easy to operate [21,22]. Due to the loading capacity of the adsorbed materials in the QCM platform being larger than the cantilever platform, the measurement’s relative error was smaller [23,24].

## 2. Materials and Methods

The adsorption and desorption capacity of MOFs for CO_2_ is dependent on many factors, such as the ligand group, central ion, morphology and size [25,26]. In this study, we employed ZIF-8, UiO-66 and MOF-74 as the research objects.

### 2.1. Material

All of the reagents were commercially available and used without any purification. Zinc nitrate hexahydrate (Zn(NO_3_)_2_·6H_2_O, 99.0%), zirconium tetrachloride (ZrCl_4_, 99.5%) and magnesium nitrate hexahydrate (Mg(NO_3_)_2_·6H_2_O, 99.0%) were obtained from Alfa Aesar. Dimethylformamide (DMF, 99.0%), methanol (CH_3_OH, 99.9%), acetone (CH_3_COCH_3_, 99.5%), ethanol (C_2_H_5_OH, 99.8%) and glacial acetic acid (CH_3_COOH, 99.99%) was purchased from Shanghai Chemical Reagent Co., Ltd (No. 52, Ningbo Road, Shanghai, China). 2,5-Dihydroxyterephthalic acid (99.0%), 2,5-dimethylterephthalic acid (99.0%) and 2-methylimidazole (99.0%) were obtained from Maclin. Carbon dioxide (CO_2_, 99.99%) was purchased from Shanghai Shenkai Co., Ltd. (No. 1769, Puxing Road, Shanghai, China).

### 2.2. Synthesis of ZIF-8

First, 5.95 g Zn(NO_3_)_2_·6H_2_O (0.020 mol) and 6.16 g 2-methylimidazole (0.075 mol) were each dissolved into 150 mL methanol. Then, the ligand solution was added to the zinc salt solution under stirring for ten minutes at 298 K. After stirring for 24 h, the precipitate was separated and washed with 50 mL methanol several times. Finally, the product was dried by critical point dryers (SCD-350M, Shianjia Tech. Building 1, Shuangheli, Huangcun Town, Beijing, China).

### 2.3. Synthesis of UiO-66

First, 70 mg ZrCl_4_ (0.3 mmol) and 30 equivalents of glacial acetic acid (9 mmol) were dissolved into 10 mL DMF under ultrasound. Then, 58 mg 2,5-dimethylterephthalic acid (0.3 mmol) was added to the zirconium salt solution under ultrasound at 298 K. Next, the obtained mixture was transferred into a Teflon-lined stainless-steel autoclave (25 mL) and heated in an electric oven at 393 K for 24 h. The precipitate was taken out and washed several times with DMF to remove any unattached products. Finally, the product was dried using a supercritical dryer.

### 2.4. Synthesis of MOF-74

First, 239 mg 2,5-dihydroxyterephtalic acid (1.20 mmol) and 928 mg Mg(NO_3_)_2_·6H_2_O (3.62 mmol) were each dissolved into 30 mL DMF. Then, the acid solution was added to the magnesium salt solution under stirring for ten minutes at 298 K. After stirring for 24 h, the precipitate was separated and washed with 20 mL DMF several times. Finally, the product was dried using a supercritical dryer.

### 2.5. Fabrication of QCM Sensors and Measuring Equipment

**Scheme 1:**
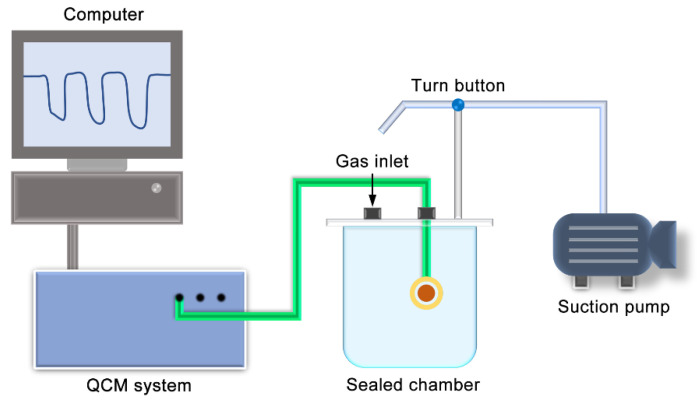
Diagrammatical illustration of the QCM sensing test platform.

The QCM adsorption measurements were performed on a modified setup that we reported previously [27]. Scheme 1 provides an illustration of the experimental setup.

Before the absorbent material was dropped onto the surface of the quartz crystal, the quartz crystal was cleaned to ensure that the sensitive material was tightly bonded to the quartz crystal. The QCM electrodes were ultrasonically cleaned in acetone, ethanol and deionized water for 30 min and then dried at 333 K. Thereafter, 2, 4, 6 and 8 μL of the suspension (material mass: deionized water volume = 1 mg:1 mL) were drop-cast onto the QCM electrode to form a sensitive film under drying at 298 K and recorded as QCM-2, QCM-4, QCM-6 and QCM-8. In this measurement, the MOF/QCM sensor was installed in a sealed chamber (0.05 L) at a different temperature. The chamber was evacuated to below 0.01 MPa before the different volumes of CO_2_ were injected. Then, the CO_2_ molecules could be adsorbed onto the surface of the MOF/QCM sensor, which induced a shift in the resonance frequency of the MOF/QCM sensor. These data were recorded by a personal computer. After each measurement, the chamber had to be evacuated to remove the CO_2_ molecules on the surface of the sensor until the frequency changed back to its initial value. Repeating the above operation, the adsorption capacity of CO_2_ under different partial pressures at a certain temperature could be obtained. In this experiment, two adsorption isotherms at different temperatures were obtained by changing the test temperature, and the heat of the adsorption was calculated using the Clausius–Clapeyron relation.

It is known that the adsorption capacity increases with a decrease in the temperature. Therefore, for the adsorption tests of the three materials, a low temperature of 273 K was adopted as the standard, and for the high temperature, a value with a larger change in the adsorption capacity was generally adopted. For the three different materials (i.e., ZIF-8, MOF-74 and UIO-66), the values for the high temperature were 313, 293 and 298 K, respectively. The whole experimental process was completed under the condition of a relative humidity of 40%. In fact, since the gas used for the whole test process was absolutely dry CO_2_ and the test chamber was a vacuum, the humidity did not affect the results of the entire experiment.

### 2.6. Characterization

A field emission scanning electron microscope (FE-SEM, JSM-6700F) was employed to observe the MOFs’ morphologies and microstructures. The powder X-ray diffraction patterns of the samples were recorded at 0.02°/s and operated at a 40 kV and 15 mA current with Cu Kα_1_ radiation (λ = 0.15406 nm) using a diffractometer (XRD, Dmax 2500V). The obtained CO_2_ adsorption–desorption isotherms were evaluated by a Micromeritics ASAP 2020 system.

### 2.7. Theoretical Calculation

The adsorption enthalpy of the CO_2_ in the ZIF-8 was simulated by Gaussian 09 calculation software. This method was referred to in previous reports [28,29]. Firstly, the minimum repetitive unit containing the crystal structure information in the ZIF-8 was intercepted, and then the structural optimization and vibration frequency analysis were carried out to confirm that there was no imaginary frequency in the structure. The same optimization was then carried out for the CO_2_ molecules. There were two sites contained in the ZIF-8 structural unit after the analysis: metal ion and nitrogen atom. From the model, the CO_2_ adsorption near the Zn^2+^ ion will have a large spatial steric resistance. After comprehensive consideration, a CO_2_ molecule was placed near the nitrogen atoms and then left to conduct a structural optimization to determine the location with the lowest energy. The adsorption enthalpy is calculated as:

$$\Delta H=H_{CO_{2}+ZIF-8}-H_{CO_{2}}-H_{ZIF-8}$$

The input (material-input.gjf) and result (material-output.out) files from the calculation can be found in the Supplementary Materials.

## 3. Results

### 3.1. Characterization of the MOFs

**Figure 1:**
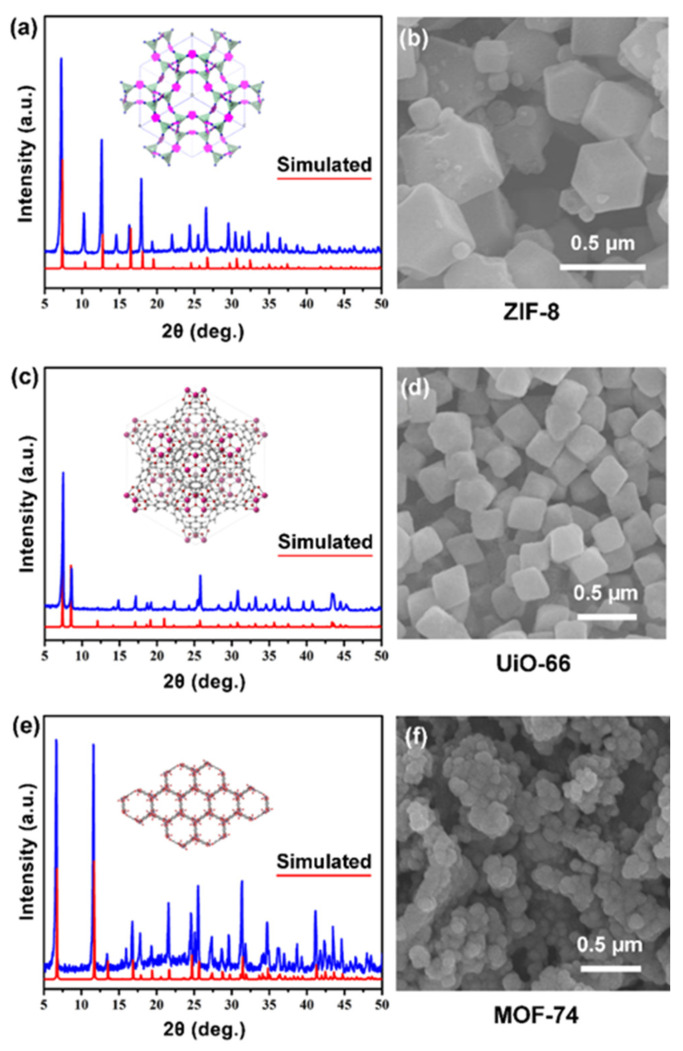
XRD experimental and simulated patterns: (**a**) ZIF-8; (**c**) UiO-66; (**e**) MOF-74. SEM images: (**b**) ZIF-8; (**d**) UiO-66; (**f**) MOF-74.

As shown in Figure 1, the patterns of the three kinds of MOFs are in good agreement with the simulated signals through the crystal information files. After observing the SEM images, the ZIF-8 had a rhombic dodecahedral shape with an average size of 500 nm, while the UiO-66 was an octahedron with a size of 250 nm, and the MOF-74 was approximately 100 nm without well-defined shapes. In order to verify whether our new method was affected by the different material factors, we selected these three MOFs with various properties, morphologies and sizes as the research objects.

### 3.2. Sensing Performance of MOF-Based QCM Sensors

After loading ZIF-8 onto QCM sensor, there is a linear relationship between the decrease of quartz crystal vibration frequency and the mass of material loaded. We can calculate the active material loading on the substrate using Equation (1) [30]:

(1) $$\Delta f=\frac{2f_{0}^{2}}{A\sqrt{\mu \rho }}\Delta m=-c\Delta m$$

where *f*_0_ (Hz) is the fundamental resonant frequency, which depends on the nature of the QCM chip; *A* (cm^2^) is the area of the silver plates coated on the quartz crystal; and *μ* (dimensionless) and *ρ* (g/cm^3^) are the shear modulus and density of the quartz crystal, respectively. Meanwhile, *c* (Hz/g) is a related constant. Apart from the mentioned invariants above, the frequency shift (Δf/Hz) is proportional to the mass change of the adsorption (Δ*m*/g) on the electrode surface of the QCM.

From Table 1, we can see that the load of the MOFs on the different QCM sensors increased with the increase in the volume of the solution coated on their surfaces. The theoretical load of QCM-2, QCM-4, QCM-6 and QCM-8 was 2000, 4000, 6000 and 8000 ng, respectively, while the actual loads were 1971, 4034, 5983 and 7969 ng. Hence, the theoretical load was proportional to the actual solution used, and the negligible differences mainly came from two aspects. One was the difference among the fundamental frequencies of the various QCM substrates, and the other may be caused by the inhomogeneity of the precursor solutions. However, these two differences had little effect after the normalized calibration.

At room temperature (298 ± 2 K), a low-concentration CO_2_ can be regarded as an ideal gas, and the specific volume of the MOFs could be neglected compared with the CO_2_ gas, and then we can obtain an approximate formula for the systematic volume changes:

(2) $$\Delta v=v_{g}\left(1-\frac{v_{c}}{v_{g}}\right)\approx v_{g}=RT/P$$

It can be obtained by substituting it into the Clausius–Clapeyron relation:

(3) $$\frac{dP}{dT}=\frac{\Delta H}{T\cdot \Delta v}$$

(4) $$\frac{dP}{dT}=\frac{P\cdot \Delta H}{T^{2}R}$$

Because the change in the adsorption enthalpy at different temperatures was very small, except for the occurrence of the reaction, it can be considered as a constant value in the calculation. Then, we get the following formulas:

(5) $$\frac{dP}{P}=\frac{\Delta H}{R}\cdot \frac{dT}{T^{2}}$$

(6) $$\int_{P_{1}}^{P_{2}}\frac{dP}{P}=\frac{\Delta H}{R}\cdot \int_{T_{1}}^{T_{2}}\frac{dT}{T^{2}}$$

(7) $$ln\frac{P_{2}}{P_{1}}=\frac{\Delta H}{R}\left(\frac{1}{T_{1}}-\frac{1}{T_{2}}\right)$$

**Figure 2:**
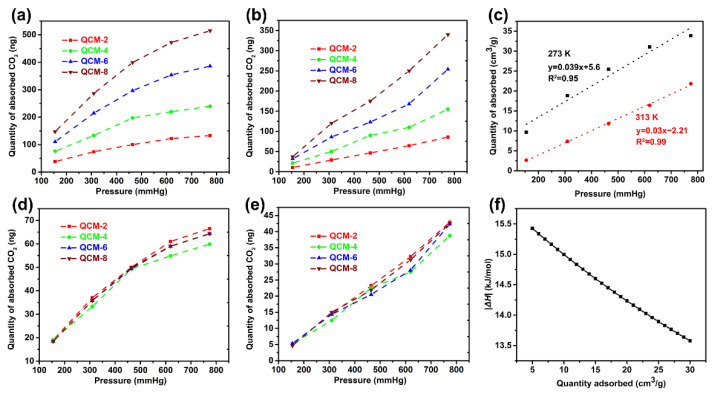
CO_2_ absorption curves of the four QCM sensors by the ZIF-8: (**a**) 273 and (**b**) 298 K. Normalized curves of the CO_2_ adsorption by the ZIF-8 sensor: (**d**) 273 and (**e**) 298 K. CO_2_ adsorption (**c**) isotherm and (**d**) enthalpy curve for the ZIF-8. (**f**) The relationship between the CO_2_ adsorption enthalpy and capacity.

Next, we tested the CO_2_ adsorption capacity of the four ZIF-8 QCM sensors at different temperatures (Figure S1), converting the frequency change value into the adsorption capacity using the previous Formula (1). The gas pressure calculated from the ideal gas equation of the state was used to obtain the results in Figure 2a,b. We can observe that the adsorption capacity of these four QCM sensors at a low temperature was far greater than at a high temperature under the same gas pressure, which could be attributed to the exothermic reaction essence of the adsorption reaction; hence, a low temperature can promote the CO_2_ adsorption reaction in this system.

The adsorption capacity of these four QCM sensors for CO_2_ gas increased obviously following the rise in the loads of the MOFs. Theoretically, the relationship between the four QCM sensors should increase in equal proportion, but there was no evidence to support this conclusion at high or low temperatures. The reason for this is that, on the one hand, a larger load will provide more adsorbate and increase its adsorption capacity, but on the other hand, because the active area on the QCM substrate is fixed, more load deposited on the same area will increase the thickness of the sensitive film, and the thickened sensitive film will lose part of the adsorption capacity, resulting in an unequal increase in the adsorption capacity. Considering the situation analyzed above, we normalized the four QCM sensors and only considered the adsorption capacity per unit of the MOFs load, resulting in Figure 2d,e. It can be seen from these two subfigures that after normalization, the adsorption capacity of the four QCM sensors was not much different, but QCM-2, which had the least load, had the highest unit adsorption capacity, while QCM-8, which had the highest load, was slightly lower than QCM-2, which shows that our previous discussion was reasonable. Based on the above analysis, we decided to select the adsorption data of QCM-2 for fitting and calculating its adsorption heat and used 2 µL of solution as the standard in the subsequent gas MOF experiments.

Figure 2c shows the two adsorption isotherms obtained by linear fitting the normalized adsorption data of QCM-2 at 273 and 313 K. Here, linear fitting rather than curve fitting was executed, because this fitting method can carry out more concise and fast calculations. Through the two adsorption isotherm equations obtained in Figure 2c, the adsorption enthalpy curve Figure 2f could be collected by taking the change value in the different frequency (adsorption capacity) as the abscissa.

From the figures, two response isotherms were gained by linear fitting the response values of the two curves with the CO_2_ volume (Figure 2c). By taking the points of the same response frequency on the isotherm, (Δ*f*, *v*_1_) and (Δ*f*, *v*_2_) could be collected. Based on Formula (1), the same Δ*f* means the same Δ*m*, which means the same mass of adsorbed CO_2_. Then, we can make the following derivations:

(8) $$\frac{P_{1}v_{1}}{RT_{1}}=\frac{P_{2}v_{2}}{RT_{2}}$$

(9) $$\frac{P_{2}}{P_{1}}=\frac{v_{1}T_{2}}{v_{2}T_{1}}$$

(10) $$ln\frac{v_{1}T_{2}}{v_{2}T_{1}}=\frac{\Delta H}{R}\left(\frac{1}{T_{1}}-\frac{1}{T_{2}}\right)$$

The Δ*H* can be calculated using Formula (10). Hence, we obtain the Δ*H-*Δ*f* curve by taking different points, as shown in Figure 2f. At last, the Δ*H* value was calculated to be in a range from −13.6–−15.6 kJ/mol.

**Figure 3:**
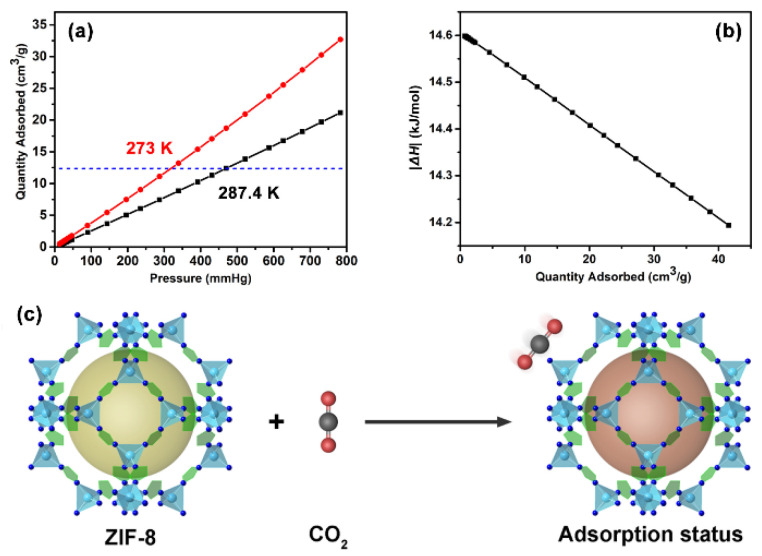
CO_2_ adsorption (**a**) isotherm and (**b**) enthalpy curve tested by ASAP2020. The (**c**) CO_2_ adsorption model of the ZIF-8.

In order to verify the reliability of our new method, we employed a large commercial adsorption instrument, ASAP2020 (Micromeritics Instrument Corporation, 4356 Communications Dr. Norcross, GA 30093-2901, U.S.A.), to measure the adsorption isotherms of the CO_2_ on the ZIF-8 (Figure 3a). By taking the same adsorption volume points, *P*_1_ and *P*_2_ at different temperatures could be gathered. Then, the data were put into Formula (7), and we obtained the Δ*H* value. After taking a series of values, we obtained a relationship curve between the Δ*H* value and the mass adsorbed CO_2_ (Figure 3b). The Δ*H* value was determined to be in the range of −14.2–−14.6 kJ/mol.

Meanwhile, the software by Gaussian was accepted to simulate the energy changes after the CO_2_ was adsorbed by the ZIF-8 (Figure 3c). The simulation and calculation were conducted using the Gaussian 09 software, with the hybrid B3LYP functional and 6-31G (d, p) basis set. The optimized structures were confirmed to be the minima on the potential energy surface via vibration frequency analysis, and the energies of the free ZIF-8, CO_2_ and adsorption status are shown in Table 2 (the CIF files can be found in Supplementary Materials Section SII).

ΔH=−3026.642909−(−188.485902)−(−2838.149458)=−0.007549

ΔH=−0.007549∗2625.500 kJ/mol≈−19.82 kJ/mol

Compared with the QCM method, the test results of the commercial adsorption instrument were closer to the simulation calculation results and had higher accuracy, but the ASAP instrument was not only bulky and expensive but also more complicated to operate and required professional operators to analyze. By comparing the Δ*H* of the QCM platform and ASAP2020, we calculated the relative error of this novel method to be 6.8%. The errors were within a small range, proving that this novel method is sufficiently reliable. The QCM method can quickly and easily obtain similar results with only a small loss of accuracy, which is exactly what the QCM method proposes.

**Figure 4:**
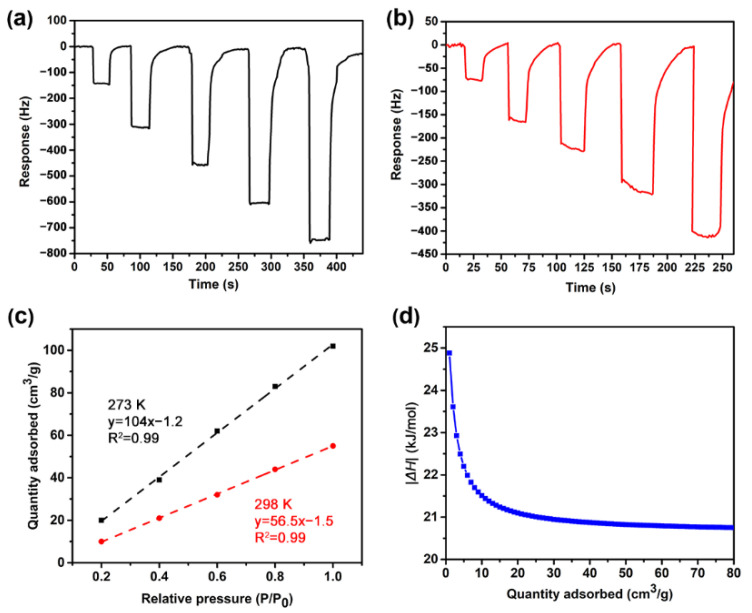
CO_2_ absorption curves of the UiO-66 QCM sensor: (**a**) 273 and (**b**) 298 K. The CO_2_ adsorption (**c**) isotherm and (**d**) enthalpy curve of the UiO-66.

In order to verify whether our new method is universal, we applied this method to other MOFs with different properties, shapes and sizes. As shown in Figure 4, the response curves of the CO_2_ on the UiO-66-based QCM platform were obtained at 273 and 298 K, respectively, and then we used the same method as the ZIF-8 for the point fitting. The adsorption enthalpy was located in the range of −24.9 and −20.8 kJ/mol, respectively, the relative error was 5.4% compared with the previous report of −25 and −22 kJ/mol [31].

**Figure 5:**
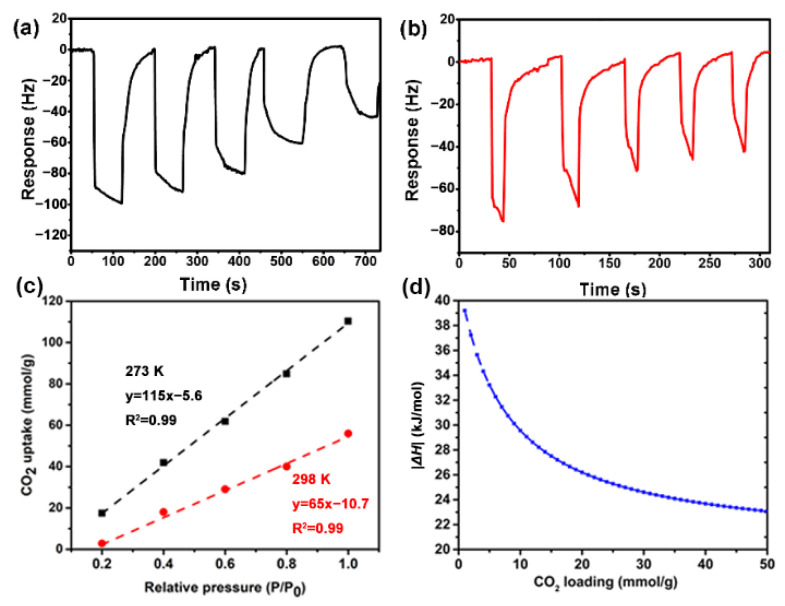
CO_2_ absorption curves of the MOF-74 QCM sensor: (**a**) 273 and (**b**) 293 K. The CO_2_ adsorption (**c**) isotherm and (**d**) enthalpy curve of the MOF-74.

Additionally, the MOF-74 was used to prove the applicability of our method in an opposite test way, as shown in Figure 5. Unlike the ZIF-8 and UiO-66, a response test from a high concentration to a low concentration was used for the MOF-74-based QCM platform. By taking the same fitting mode, we obtained Δ*H* = −39.7–−23.8 kJ/mol. Taking the previous work as a reference (−40–−25 kJ/mol), the relative error was 4.8%, which is still a small value [32].

## 4. Conclusions

In summary, we illustrated a minimalist method to extract the CO_2_ adsorption enthalpy on the surface of MOFs based on a QCM platform. The relative errors were at a very low level. The advantages of low costs, simple operation and time savings were featured. This novel method maintained a high accuracy regardless of the properties, morphology and size of the MOFs and operating habits. Based on this method for calculating gas adsorption enthalpy, researchers can find suitable CO_2_ capture or sensing materials fast and easily. In other words, the work of this paper provides a new tool for adsorption enthalpy tests using only a limited amount of material (less than 1 mg); it has simple operations (without professional operation skills) and concise calculations (without complex models), which could be comparable to the experimental results obtained from those complex and professional instruments. In addition, using the same principle, it can be extended to other gas capture technologies, implying a broad application prospect.

## Figures and Tables

**Table 1:** The MOF mass deposited on the active region of the QCM sensors.

Sample	*f*_0_ (Hz)	*c*	Δ*f* (Hz)	Δ*m* (ng)
QCM-2	9,984,966	1.82478	3595 ± 22	1971 ± 12
QCM-4	9,985,364	1.82493	7358 ± 18	4034 ± 10
QCM-6	9,985,096	1.82483	10,912 ± 15	5983 ± 8
QCM-8	9,984,417	1.82458	14,535 ± 24	7969 ± 13

**Table 2:** Simulated energies of the CO_2_ adsorbed by ZIF-8.

Item	CO_2_	ZIF-8	CO_2_ + ZIF-8
H (Hartree)	−188.485902	−2838.149458	−3026.642909
E (Hartree)	−188.489581	−2838.176612	−3026.674564
H-correction	0.014368	0.378423	0.394279
E-correction	0.010688	0.351269	0.362624

## Data Availability

Not applicable.

## Supplementary Materials

The following supporting information can be downloaded at: https://www.mdpi.com/article/10.3390/nano12224035/s1, Figure S1: The CO_2_ response curves of different QCM sensors at (a) 313 K and (b) 273 K. Attached calculation files: S01 Simulation structure and energy of ZIF-8; S02 Simulation structure and energy of CO_2_ molecule; S03 Simulated adsorption of ZIF-8 to CO_2_ and calculated enthalpy; S04 Archive file of calculation input and output data.

## References

[1] Sol D., Laca A., Laca A., Díaz M. (2020). Approaching the environmental problem of microplastics: Importance of WWTP treatments. Sci. Total Environ..

[2] Leitão N.C. (2021). The effects of corruption, renewable energy, trade and CO_2_ emissions. Economies.

[3] Yoro K.O., Daramola M.O. (2020). CO_2_ emission sources, greenhouse gases, and the global warming effect. Advances in Carbon Capture.

[4] Ekwurzel B., Boneham J., Dalton M.W., Heede R., Mera R.J., Allen M.R., Frumhoff P.C. (2017). The rise in global atmospheric CO_2_, surface temperature, and sea level from emissions traced to major carbon producers. Clim. Chang..

[5] Treharne R., Bjerke J.W., Tømmervik H., Stendardi L., Phoenix G.K. (2019). Arctic browning: Impacts of extreme climatic events on heathland ecosystem CO_2_ fluxes. Glob. Chang. Biol..

[6] Anwar M., Fayyaz A., Sohail N., Khokhar M., Baqar M., Khan W., Rasool K., Rehan M., Nizami A. (2018). CO_2_ capture and storage: A way forward for sustainable environment. J. Environ. Manag..

[7] Xu R., Li R., Ma J., He D., Jiang P. (2017). Effect of mineral dissolution/precipitation and CO_2_ exsolution on CO_2_ transport in geological carbon storage. Acc. Chem. Res..

[8] Ren G., Ma G., Cong N. (2015). Review of electrical energy storage system for vehicular applications. Renew. Sustain. Energy Rev..

[9] Gao D., Arán-Ais R.M., Jeon H.S., Roldan Cuenya B. (2019). Rational catalyst and electrolyte design for CO_2_ electroreduction towards multicarbon products. Nat. Catal..

[10] Thompson W.A., Sanchez Fernandez E., Maroto-Valer M.M. (2020). Review and analysis of CO_2_ photoreduction kinetics. ACS Sustain. Chem. Eng..

[11] Zhu Q. (2019). Developments on CO_2_-utilization technologies. Clean Energy.

[12] Waheed R., Chang D., Sarwar S., Chen W. (2018). Forest, agriculture, renewable energy, and CO_2_ emission. J. Clean. Prod..

[13] Li X., Yao D., Wang D., He Z., Tian X., Xin Y., Su F., Wang H., Zhang J., Li X. (2022). Amino-functionalized zifs-based porous liquids with low viscosity for efficient low-pressure CO_2_ capture and CO_2_/N_2_ separation. Chem. Eng. J..

[14] Ahmed R., Liu G., Yousaf B., Abbas Q., Ullah H., Ali M.U. (2020). Recent advances in carbon-based renewable adsorbent for selective carbon dioxide capture and separation-a review. J. Clean. Prod..

[15] Thallapally P.K., Peter McGrail B., Dalgarno S.J., Schaef H.T., Tian J., Atwood J.L. (2008). Gas-induced transformation and expansion of a non-porous organic solid. Nat. Mater..

[16] Avci G., Erucar I., Keskin S. (2020). Do new MOFs perform better for CO_2_ capture and H_2_ purification? Computational screening of the updated mof database. ACS Appl. Mater. Interfaces.

[17] Yu J., Xie L.-H., Li J.-R., Ma Y., Seminario J.M., Balbuena P.B. (2017). CO_2_ capture and separations using mofs: Computational and experimental studies. Chem. Rev..

[18] Ghanbari T., Abnisa F., Daud W.M.A.W. (2020). A review on production of metal organic frameworks (MOF) for CO_2_ adsorption. Sci. Total Environ..

[19] Tuantranont A., Wisitsora-At A., Sritongkham P. (2011). A review of monolithic multichannel quartz crystal microbalance: A review. Anal. Chim. Acta.

[20] Alassi A., Benammar M., Brett D. (2017). Quartz crystal microbalance electronic interfacing systems: A review. Sensors.

[21] Xu P., Xu T., Yu H., Li X. (2017). Resonant-gravimetric identification of competitive adsorption of environmental molecules. Anal. Chem..

[22] Xu P., Yu H., Guo S., Li X. (2014). Microgravimetric thermodynamic modeling for optimization of chemical sensing nanomaterials. Anal. Chem..

[23] Xu T., Xu P., Zheng D., Yu H., Li X. (2016). Metal–organic frameworks for resonant-gravimetric detection of trace-level xylene molecules. Anal. Chem..

[24] Yu H., Yang T., Chen Y., Xu P., Lee D.-W., Li X. (2012). Chemo-mechanical joint detection with both dynamic and static microcantilevers for interhomologue molecular identification. Anal. Chem..

[25] Stock N., Biswas S. (2012). Synthesis of metal-organic frameworks (MOFs): Routes to various mof topologies, morphologies, and composites. Chem. Rev..

[26] Qian Y., Zhang F., Pang H. (2021). A review of MOFs and their composites-based photocatalysts: Synthesis and applications. Adv. Funct. Mater..

[27] Ma Z., Yuan T., Fan Y., Wang L., Duan Z., Du W., Zhang D., Xu J. (2020). A benzene vapor sensor based on a metal-organic framework-modified quartz crystal microbalance. Sens. Actuators B Chem..

[28] Wilmer C.E., Farha O.K., Bae Y.-S., Hupp J.T., Snurr R.Q. (2012). Structure–property relationships of porous materials for carbon dioxide separation and capture. Energy Environ. Sci..

[29] Wang L., Wang Z., Xiang Q., Chen Y., Duan Z., Xu J. (2017). High performance formaldehyde detection based on a novel copper (II) complex functionalized QCM gas sensor. Sens. Actuators B Chem..

[30] Wang L. (2020). Metal-organic frameworks for qcm-based gas sensors: A review. Sens. Actuators A Phys..

[31] Huang Y., Qin W., Li Z., Li Y. (2012). Enhanced stability and CO_2_ affinity of a UiO-66 type metal–organic framework decorated with dimethyl groups. Dalton Trans..

[32] Yang D.-A., Cho H.-Y., Kim J., Yang S.-T., Ahn W.-S. (2012). CO_2_ capture and conversion using Mg-MOF-74 prepared by a sonochemical method. Energy Environ. Sci..

