# Supplementary material for: New Application of Quartz Crystal Microbalance: A Minimalist Strategy to Extract Adsorption Enthalpy
Source: Nanomaterials (Basel). 2022 Nov 17;12(22):4035. doi: 10.3390/nano12224035 (PMC9693904; doi:10.3390/nano12224035)
Supplement: Supplementary file 1 [file nanomaterials-12-04035-s001.zip › nanomaterials-2011654-supplementary.pdf]

Supplementary Materials

New Application of Quartz Crystal Microbalance: A Minimalist Strategy to Extract Adsorption Enthalpy

Zhiheng Ma, Tongwei Yuan *, Yu Fan, Yang Chen, Yueling Bai, Zhixuan Cheng and Jiaqiang Xu *

NEST Laboratory, Department of Physics, Department of Chemistry, College of Science, Shanghai University, Shanghai 200444, China

* Correspondence: twkiller@outlook.com (T.Y.); xujiaqiang@shu.edu.cn (J.X.)

Section I.

Supporting figures

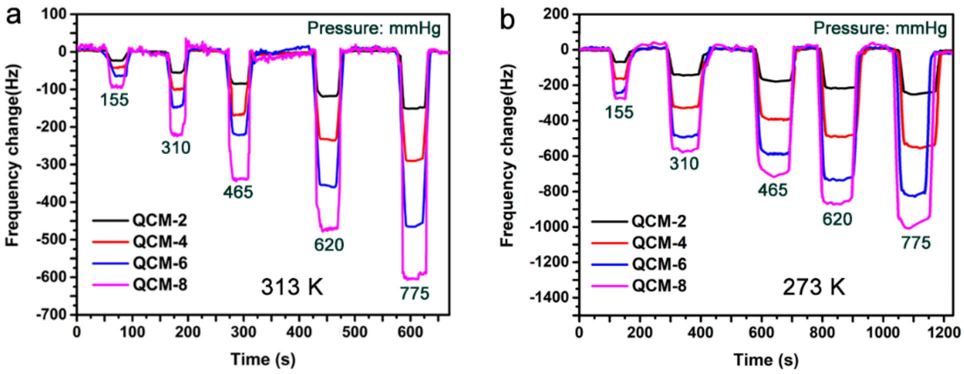

Figure S1. The CO_2 response curves of different QCM sensors at (a) 313 K and (b) 273 K.

Section II

Attached files of simulated calculations (Gaussian 09)

ZIF-8.LOG

File S1. Simulation structure and energy of ZIF-8.

CO2.LOG

File S2. Simulation structure and energy of CO_2 molecule.

ZIF-8+CO2.LOG

File S3. Simulated adsorption of ZIF-8 to CO_2 and calculated enthalpy.
